# Intravital correlated microscopy reveals differential macrophage and microglial dynamics during resolution of neuroinflammation

**DOI:** 10.1242/dmm.014886

**Published:** 2014-07

**Authors:** Tjakko J. van Ham, Colleen A. Brady, Ruby D. Kalicharan, Nynke Oosterhof, Jeroen Kuipers, Anneke Veenstra-Algra, Klaas A. Sjollema, Randall T. Peterson, Harm H. Kampinga, Ben N. G. Giepmans

**Affiliations:** 1 Department of Cell Biology, University Medical Center Groningen, University of Groningen, A. Deusinglaan 1, 9713 AV, Groningen, The Netherlands.; 2 Massachusetts General Hospital, Harvard Medical School, 149 13th Street, Charlestown, MA 02129, USA.

**Keywords:** Brain, Intravital microscopy, Leukocytes, Microglia, Neurodegeneration, Zebrafish

## Abstract

Many brain diseases involve activation of resident and peripheral immune cells to clear damaged and dying neurons. Which immune cells respond in what way to cues related to brain disease, however, remains poorly understood. To elucidate these *in vivo* immunological events in response to brain cell death we used genetically targeted cell ablation in zebrafish. Using intravital microscopy and large-scale electron microscopy, we defined the kinetics and nature of immune responses immediately following injury. Initially, clearance of dead cells occurs by mononuclear phagocytes, including resident microglia and macrophages of peripheral origin, whereas amoeboid microglia are exclusively involved at a later stage. Granulocytes, on the other hand, do not migrate towards the injury. Remarkably, following clearance, phagocyte numbers decrease, partly by phagocyte cell death and subsequent engulfment of phagocyte corpses by microglia. Here, we identify differential temporal involvement of microglia and peripheral macrophages in clearance of dead cells in the brain, revealing the chronological sequence of events in neuroinflammatory resolution. Remarkably, recruited phagocytes undergo cell death and are engulfed by microglia. Because adult zebrafish treated at the larval stage lack signs of pathology, it is likely that this mode of resolving immune responses in brain contributes to full tissue recovery. Therefore, these findings suggest that control of such immune cell behavior could benefit recovery from neuronal damage.

## INTRODUCTION

In the vertebrate brain, immune cells including microglia can play both beneficial and detrimental roles in response to, for example, traumatic injury, stroke or neurodegenerative disease (Dirnagl et al., 1999; Shechter and Schwartz, 2013a; Sokolowski and Mandell, 2011). A supportive function can be mediated by clearing dead cells and debris, and possibly disease-related products such as protein aggregates (London et al., 2013; Mildner et al., 2011; Shechter and Schwartz, 2013b; Sokolowski and Mandell, 2011). Such a protective function of immune cells might be lost due to aging and could thereby contribute to, for example, Alzheimer’s disease (Streit et al., 2009). By contrast, disease phenotypes can be perpetuated by infiltration of neutrophils in stroke or by overactivation of microglia, causing chronic inflammatory responses (Brown and Neher, 2012; Meda et al., 1995; Zhang et al., 2005). Nevertheless, following brain damage it remains unclear which immune cells are recruited under what circumstances; how these immune cells disappear again; and how resolution of neuroinflammation is achieved (Schwartz and Baruch, 2014).

Microglia are self-renewing resident immune cells migrating into the brain in early embryonic development in vertebrates (Ginhoux et al., 2010; Herbomel et al., 1999; Hickey and Kimura, 1988). Inactive microglia are ramified, but when activated they become amoeboid and resemble monocyte-derived macrophages (MDMs), both in appearance as well as in function. Under particular disease or injury conditions, peripheral immune cells, including MDMs, can traffic to the central nervous system, possibly by migrating through blood vessels, through the meninges that envelope the brain or via the brain-ventricular choroid plexus (Ransohoff et al., 2003; Shechter et al., 2013). Due to a lack of proper selective markers as well as heterogeneity of macrophages and microglia, macrophages have often been mistaken for activated microglia (Prinz et al., 2011). This has precluded distinguishing the identities and roles of specific immune cell subclasses, in particular macrophages and microglia, in conditions affecting the brain.

Following recruitment and/or activation of immune cells, phagocytosis is a key response to tissue damage, ensuring control of inflammation and tissue repair (Nagata et al., 2010; Ravichandran and Lorenz, 2007; Savill et al., 2002). In peripheral tissue, cells undergoing programmed cell death are cleared by macrophages in an anti-inflammatory process (Nagata et al., 2010; Ravichandran and Lorenz, 2007; Savill et al., 2002). Clearance is followed by a termination phase, known as resolution of inflammation, in which immune cells that are no longer needed succumb to programmed cell death or exit through lymphatic vessels (Serhan and Savill, 2005). In the brain, however, it is unclear when and which types of immune cells are recruited for clearance and how resolution of inflammation occurs. Analogous to peripheral immune responses, in the brain these responses show distinct phases and the nature of the primary response after insult differs from that at later times (Gelderblom et al., 2009).

The first *in vivo* live imaging studies on microglia performed in mice revealed that, unexpectedly, microglia under physiological conditions are highly dynamic (Davalos et al., 2005; Nimmerjahn et al., 2005; Tremblay et al., 2011). Therefore, detailed temporal resolution analysis of immune cells is needed to understand immune responses under pathological conditions. Animal models for stroke by ischemia do allow a temporal analysis of infiltrating and resident leukocytes, and have shown infiltration of neutrophils, monocytes, as well as locally recruited resident microglia (Davies et al., 1998; Gelderblom et al., 2009). In this case, neutrophils increase the damage after ischemia, as blockage of their entry halts damage in mouse models (Dirnagl et al., 1999). To identify the relevant immune cells and resolve spatiotemporal aspects of neuroinflammation in vertebrates, we have induced genetically targeted cell death in the zebrafish brain as a proxy for neurodegeneration. Larval zebrafish are transparent and allow non-invasive intravital imaging of neurodegeneration and leukocyte behavior (Renshaw and Trede, 2012; van Ham et al., 2010). Furthermore, they have proven to be a great tool for functional genomics and drug discovery (Hwang et al., 2013; Zon and Peterson, 2005). Although many zebrafish counterparts of mammalian immune mediators remain to be identified, recent studies have revealed zebrafish homologs of factors controlling macrophage behavior (Zakrzewska et al., 2010).

**TRANSLATIONAL IMPACT**

**Clinical issue**

During stroke, neurodegeneration and many other brain diseases, the microglia (self-renewing immune cells that are resident in the brain) and peripheral immune cells such as monocyte-derived macrophages are activated to clear damaged and dying neurons. Clearance is followed by a termination phase, known as resolution of inflammation, in which immune cells that are no longer needed succumb to programmed cell death or exit the brain through lymphatic vessels. Currently, it is unclear which immune cells are involved at what stage of the disease process, and whether this response is beneficial or detrimental because macrophages and activated microglia are heterogeneous and a lack of selective markers has precluded the detailed study of their roles in conditions affecting the brain.

**Results**

Here, the authors use intravital microscopy of cellular interactions in living zebrafish brain and electron microscopy to provide new insights into the immune response to brain injury, and to determine how neuroinflammation is stopped *in vivo*. The authors use a previously established model to ablate brain cells in which transgenic nitroreductase enzyme causes cell death in targeted cells in the presence of a ligand. Using intravital imaging, they show that peripheral macrophages and resident microglia are both involved in clearing dying cells. In addition, they use electron microscopy to provide an unprecedented view of the cellular and ultrastructural features associated with neuronal ablation and the subsequent immune responses. Notably, they show that the timing of macrophage and microglia involvement is different, with macrophages being present at early stages whereas microglia dominate several days after the ablation. Other immune cells, including granulocytes, are not involved in the immune response. Finally, they show that after an initial increase, the numbers of immune cells in the brain decline, in part through phagocytes becoming apoptotic and being cleared by activated microglia.

**Implications and future directions**

These findings show that intravital microscopy in zebrafish can be used to discover immune maintenance mechanisms in the brain that would be missed using other approaches. Most surprisingly, they show that brain phagocytes undergo apoptosis and are engulfed by microglia, which suggests that inflammation in the brain can be resolved in a way similar to peripheral wounding responses, but that different immune cell types are involved. These novel findings could provide a handle for modifying the toxic, chronic neuroinflammatory processes that are found in many brain diseases.

Our model provides a platform to study the dynamics of neuroinflammatory responses *in vivo* and how these are initiated and terminated. We find that dying neurons are initially effectively cleared by microglia and non-resident macrophages and subsequently by microglia, without involvement of infiltrating neutrophils or resident astrocytes. During the neuroinflammatory resolution phase, macrophage and microglia numbers decrease by exiting the central nervous system, and programmed cell death is followed by their phagocytosis by microglia.

## RESULTS

### Targeted ablation induces a phagocytic response

**Fig. 1.**
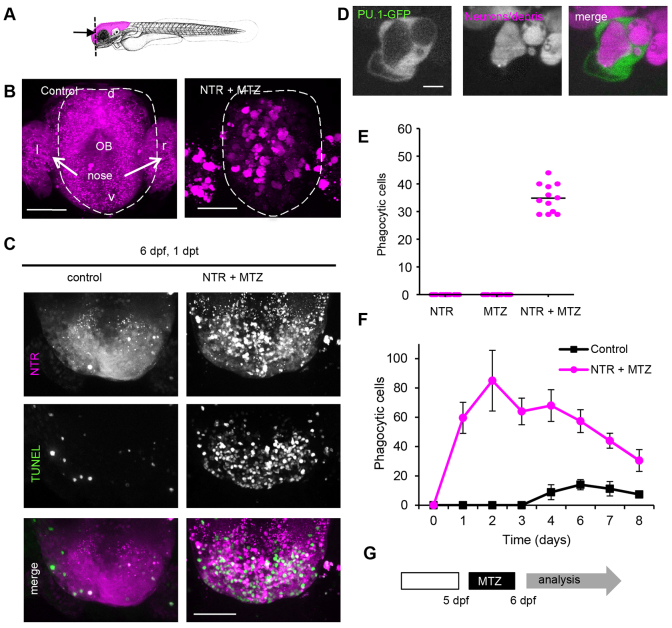
**Controlled ablation of brain cells is followed by phagocytosis and functional tissue recovery.** (A) Mid-sagittal schematic indicating NTR and fluorescent transgene expression in the brain. (B) Frontal fluorescent images of forebrain, including tip of olfactory bulb and olfactory epithelium, showing expression of fluorescent protein in brain cells expressing NTR in control animals (non-treated) and phagocytosed fluorescent protein in phagocytic cells in 3-day-old MTZ-treated animals 1 day post-treatment. (C) Dorsal images of anterior tip of forebrain in 6-day-old control larvae and larvae treated at 5 dpf, showing TUNEL-marked apoptotic cells in green. Animals were treated overnight with 2 mM MTZ at 5 dpf. (D) Phagocytic cell marked by mCherry-positive phagosome expressing PU.1-driven GFP in cytoplasm in 3 dpf animals. (E) Number of phagocytic cells in the forebrain 1 day after ablation in untreated 6 dpf NTR animals, control animals treated with MTZ and NTR animals treated with MTZ (*n*=12). Phagocytic cells were quantified by counting bright-fluorescently marked phagocytes, which can be distinguished from neurons showing low fluorescence, in the forebrains of treated and control animals. (F) Phagocytic cells in whole brain of control (untreated) and NTR animals treated with MTZ, 1–8 days post-treatment (*n*=8). Animals were treated at 5 dpf. (G) MTZ treatment regime causing brain cell death, phagocytic response and functional recovery (supplementary material Fig. S1). Experimental sequence used in C, E, F and rest of manuscript: 5-day-old pdf larvae were treated with MTZ for 16 hours followed by analysis. l and r indicate left and right; d and v, indicate dorsal and ventral; OB, olfactory bulb. Scale bars: 50 μm (B,C), 5 μm (D). See also supplementary material Fig. S1 and Movie 1.

To address the nature and kinetics of leukocyte recruitment in response to cell death in the brain, we established a model system that allows controlled ablation of neurons: nitroreductase (NTR)-mediated cell killing in zebrafish larval brain (Fig. 1A–C) (van Ham et al., 2012). Targeted expression of the bacterial enzyme NTR in combination with addition of metronidazole (MTZ) is used to induce ablation of specific tissues in zebrafish (Curado et al., 2007; Montgomery et al., 2010). The fate of ablated neurons can be monitored by analyzing the mCherry fluorescent signal, which is fused to neuronal-targeted NTR (Fig. 1B–D). We previously showed that upon engulfment of dead neurons, fluorescent mCherry accumulates in phagocytic vacuoles inside phagocytic leukocytes (van Ham et al., 2012). Because of the locally high levels of fluorescence inside these vacuoles, they can be distinguished from neurons that typically show much lower levels of cytoplasmic and nuclear fluorescence, and in addition exhibit a very different morphology.

A single overnight pulse of MTZ led to dose-dependent programmed cell death in 3-day-old fish larvae expressing neuronal NTR in the brain, but not in control zebrafish (Fig. 1B). Following neuronal cell ablation, the red cell corpses were actively taken up by phagocytes (Fig. 1B,D) (van Ham et al., 2012). This strong phagocytic response was only observed in degenerative brain regions of MTZ-treated NTR-expressing transgenic larvae, and only a few of these were seen in control animals. To identify whether these cells are PU.1-expressing primitive macrophages, the NTR larvae expressing PU.1-driven green fluorescent protein (GFP) were analyzed following ablation. The red fluorescent (dying) cells were taken up by GFP-positive phagocytes, indicating that primitive macrophages are involved in phagocytosis at this early developmental stage (Fig. 1D).

### Ablated larvae recover from ablation and grow to adulthood

To further define the roles of other immune cells, animals were studied later in development, when microglia and other immune cells have developed (Herbomel et al., 2001; Peri and Nüsslein-Volhard, 2008; Renshaw et al., 2006) (Fig. 1C,E,F; supplementary material Movie 1). In 5-day-old animals expressing neuro-NTR, MTZ also induced dose-dependent larval death (Fig. 1C; supplementary material Movie 1). Relatively low doses of MTZ (2 mM) resulted in high levels of neuronal cell death and many fluorescently marked phagocytic cells not found in control larvae (Fig. 1C; supplementary material Movie 1). However, over 80% of animals survived for over two weeks and grew to adulthood (supplementary material Fig. S1).

To address how the immune response develops in such animals, phagocytes were counted on the basis of highly fluorescent phagosomes in the forebrains of animals at different stages after ablation. The fluorescent mCherry protein (Shaner et al., 2008) is highly stable and thus allows the study of temporal aspects of immune cell clearance over the course of several days. The number of phagocytic cells initially increased to reach a maximum at around 2 days after induction of cell death, returning to near basal levels one week post-treatment (Fig. 1E,F). Ablation did not grossly affect the size, morphology and behavior of the fish, most of which survived to adulthood. Furthermore, adults that received neuronal ablation at larval stages showed normal vital functions, including feeding and mating behavior. To further investigate whether the brains of recovered animals showed any signs of pathology, the brains of NTR animals treated with MTZ were analyzed 28 days post-treatment. Histologic analysis of these brains showed normal neuronal nuclei and white matter and no spongy appearance or signs of cell death (see later). There was no apparent pathology at the cellular level (supplementary material Fig. S1). These results suggest that neuronal ablation is followed by a transient immune response that is not detrimental to recovery.

### Astrocytes are not involved in clearance

**Fig. 2.**
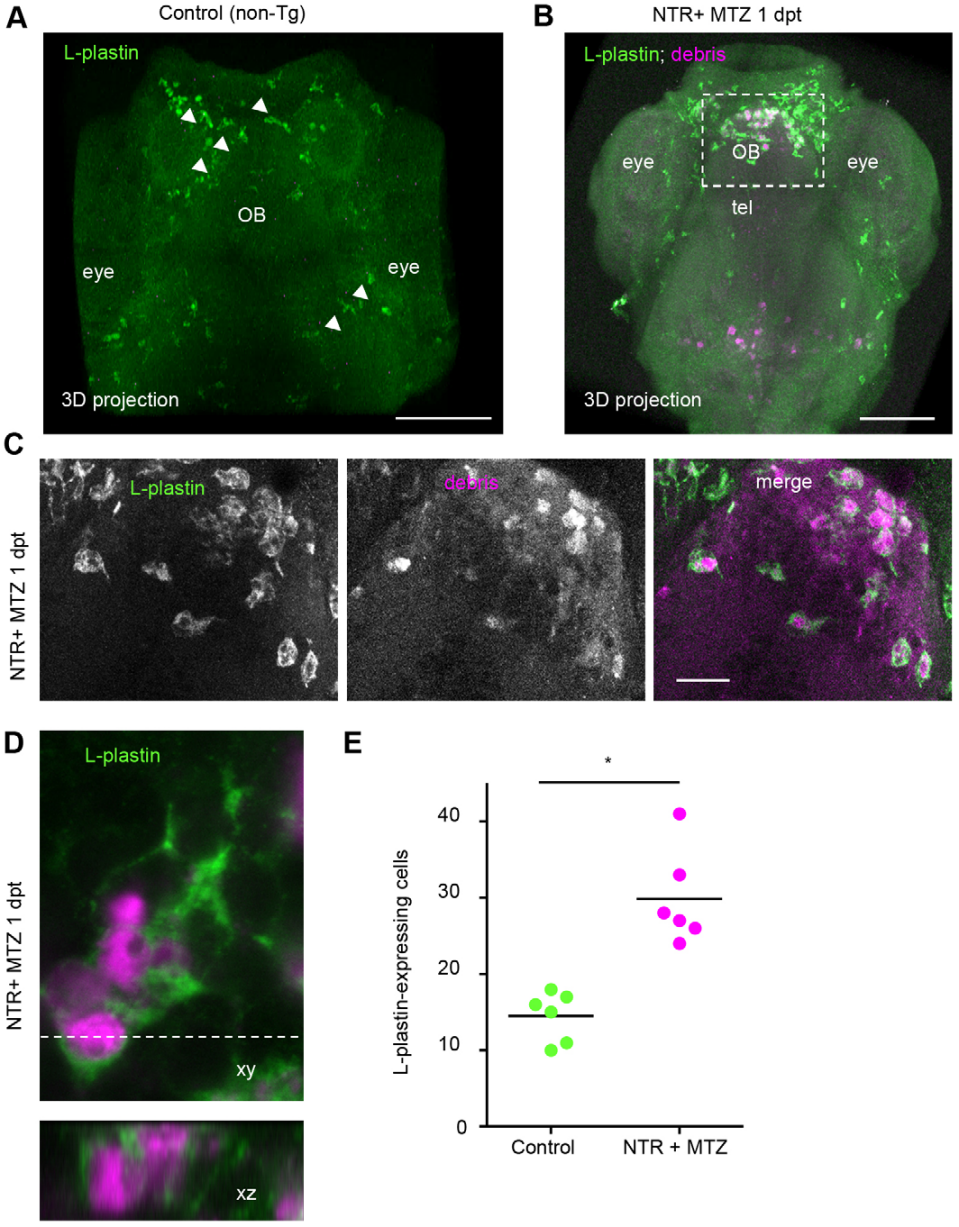
**Phagocytic cells are L-plastin-positive leukocytes.** (A,B) L-plastin fluorescent marked cells (green) in (A) whole mount stained control (non-transgenic non-treated) and (B) NTR + MTZ larvae at 1 day post-treatment (dpt). (C) L-plastin marked cells in forebrain of NTR animals treated with MTZ 1 day after treatment, showing cytoplasmic mCherry inclusions (magenta) and amoeboid morphology. (D) High magnification of L-plastin-expressing leukocyte, and orthogonal view, showing cytoplasmic inclusions of mCherry in NTR MTZ-treated animals 1 day post-treatment. (E) Quantification of L-plastin-expressing leukocytes within forebrain, in control and ablated larvae 3 days post-treatment, shows twofold increase in NTR animals treated with MTZ (*n*=6) (*P≤0.05). Z-stacks of ~80 μm were used for quantification. OB, olfactory bulb; tel, telencephalon. Scale bars: 100 μm (A,B), 20 μm (C). See also supplementary material Movie 2.

In addition to microglia, other glial cells such as astrocytes might be able to clear cellular debris (Cahoy et al., 2008). To determine whether all phagocytic cells are leukocytes, whole mount fluorescent immunostaining was performed on ablated larvae using a pan-leukocytic marker in zebrafish (L-plastin; supplementary material Table S1) (Cvejic et al., 2008). In ablated larvae, phagocytized fluorescent debris was found inside the L-plastin-expressing cells (Fig. 2B–D). No debris was found in radial glia cells, marked by glial fibrillary acidic protein (GFAP)-driven GFP (data not shown). L-plastin-expressing cells were enlarged, amoeboid and showed fewer and shorter appendages (Fig. 2D; supplementary material Movie 2). In addition to these morphological changes, the number of L-plastin-expressing cells in the forebrain region was increased twofold at 3 days after ablation (Fig. 2E).

### Neutrophils are not involved in clearance

**Fig. 3.**
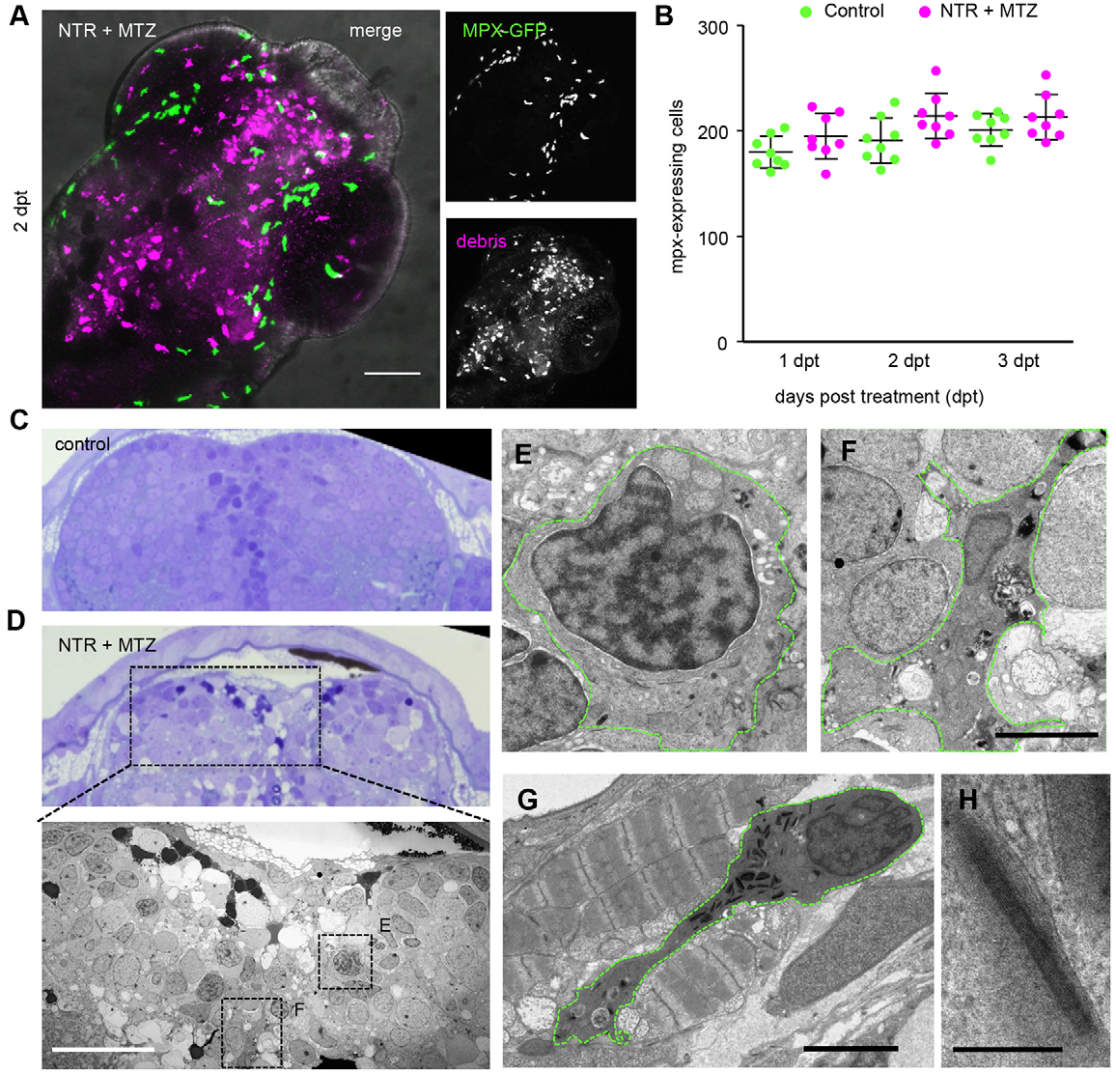
**Neutrophils are not affected by cell ablation in the brain.** (A) Myeloperoxidase (mpx)-expressing neutrophils (green) in NTR MTZ-treated brain (magenta) 2 days post-treatment. (B) Total numbers of mpx-expressing neutrophils in whole animal controls and 1–3 days post-treatment (*n*=10 animals). Numbers do not differ significantly. (C,D) Toluidine Blue stained 1 micron section of brain from control and NTR MTZ-treated animals. Control brain show homogeneous cellular profiles, whereas NTR animals treated with MTZ shows cells irregular in staining density, cytoplasmic inclusions and dark pyknotic nuclei. (D, lower panel) Electron micrograph of region marked in D shows features not found in control brains, including dark stained cells, phagocytic leukocytes and spongy appearance of tissue (also see Fig. 4). (E) High magnification of monocyte-like cell in D. (F) High magnification view of phagocytic cell in D. (G,H) Neutrophil (G) marked by cigar-shaped granules (H), located between jaw muscle cells, showing characteristic striping pattern not found in the brain. Scale bars: 50 μm (A,D, lower panel), 5 μm (F,G), 200 nm (H).

Infiltrating peripheral neutrophils could account for the increase in L-plastin-expressing cells. To address this, we performed neuronal ablation in transgenic animals expressing myeloperoxidase (mpx)-driven GFP, resulting in GFP-positive granulocytes (Renshaw et al., 2006) (supplementary material Table S1). Granulocytes are normally not found in the brain but can infiltrate the neural tissue upon injection of bacteria into brain ventricles, or upon invasive spinal cord injury (Goldshmit et al., 2012; Hall et al., 2012). However, GFP-expressing cells did not overlap with phagocytes and were not found inside the brain or spinal cord, neither immediately nor several days after ablation in neuro-NTR or control larvae (Fig. 3A; compare with Fig. 2B,C showing colocalization of mCherry and L-plastin). Granulocyte numbers were not different in MTZ-treated neuro-NTR and control larvae (Fig. 3B), suggesting the absence of increased granulopoiesis, which occurs upon systemic infection (Hall et al., 2012). In addition, no fluorescent cellular debris was found inside mpx-GFP labeled cells, suggesting that dead neurons are not engulfed by granulocytes.

To further define the nature of the phagocytic cells, ultrastructural determination was performed using electron microscopy (EM). Complete sections of forebrains of control and NTR larvae treated with MTZ were subjected to EM (Fig. 3C,D). Cells that resembled monocytes and phagocytic cells were found in the brains of ablated animals but were not present in control brains (Fig. 3E,F). Zebrafish neutrophil granulocytes are characterized by a typical nuclear morphology and by the presence of cigar-shaped crystalline granules (Lieschke et al., 2001). Although granulocytes were readily identified in zebrafish larval thin sections (Fig. 3G,H), these cells were not found in complete brain sections from control or NTR-ablated animals (Fig. 3C,D). Thus, the phagocytic cells we observed are not granulocytes, and granulocytes are not affected in behavior or number in response to controlled cell death in the nervous system. This is in contrast to reported findings following spinal cord injury or ventricle injection of bacteria in larvae (Goldshmit et al., 2012; Hall et al., 2012).

### Phagocytic cells exhibit features typical of mononuclear phagocytes

**Fig. 4.**
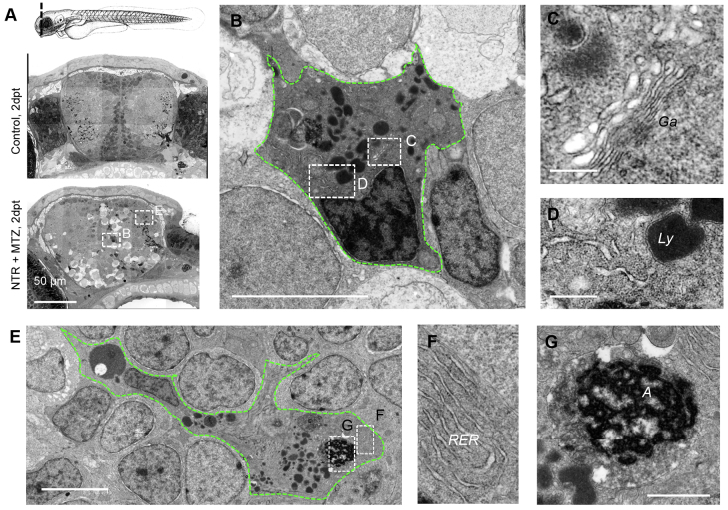
**Phagocytes with ultrastructural microglial features appear following neuronal cell death.** (A) Nanotomy of brains of 7 dpf control and NTR animals treated with MTZ 2 days post treatment, showing features specific to the NTR degenerative brain, including phagocytic leukocytes, dark cells undergoing cell death and spongy appearance of neural tissue in comparison with control. (B–D) High magnification view of phagocytic cell in A (lower panel) showing typical amoeboid microglial features including prominent Golgi apparatus (Ga; C), inclusions including lysosomal vacuoles (Ly; D) and distinctive long stretches of endoplasmic reticulum (D). (E–G) High magnification view of phagocytic cell in A showing typical amoeboid microglial cell features, including rough endoplasmic reticulum (RER; F) and engulfed cell corpse (‘A’; G). Scale bars: 50 μm (A), 200 nm (C,D), 5 μm (B,E), 1 μm (G).

To further classify the type of phagocytes in neuro-NTR animals treated with MTZ, large-scale electron microscopy (nanotomy) was performed, which allowed systematic large area analysis at nanoscale resolution (Faas et al., 2012; Ravelli et al., 2013). Nanotomy of control brain sections revealed typical ultrastructural features of neural tissue of the rostral forebrain, including neuronal nuclei, synaptic membranes, synaptic vesicles and olfactory fiber bundles (Fig. 4A). In NTR MTZ-treated larvae, phagocytes with large vacuoles were detected, which were not present in healthy animals (Fig. 4A,B,E).

Previous EM studies have identified macrophage-like and amoeboid microglia in vertebrates (Stensaas and Reichert, 1971; Tseng et al., 1983). Characteristic features of amoeboid microglia include elongated nuclei, clumps of patchy chromatin next to the nuclear envelope, prominent Golgi apparatus, free polyribosomes, granular endoplasmic reticulum (ER) with long narrow cisternae, relatively dark or dense cytoplasm and numerous inclusions such as phagosomes, lipid droplets and lysosomes. All these hallmarks, previously attributed to microglia in mammalian tissue, were also found for the phagocytic cells in ablated larvae brains (Fig. 4B–G). Although these features distinguish microglia in the healthy brain, under pathological conditions infiltrating MDMs are also characterized by these features (Ling et al., 1980; Tremblay et al., 2010; Tseng et al., 1983). Therefore, ultrastructural analysis revealed that the phagocytic cells included microglia or perhaps other mononuclear phagocytes such as MDMs.

### The ratio of ApoE-expressing microglia increases over time

**Fig. 5.**
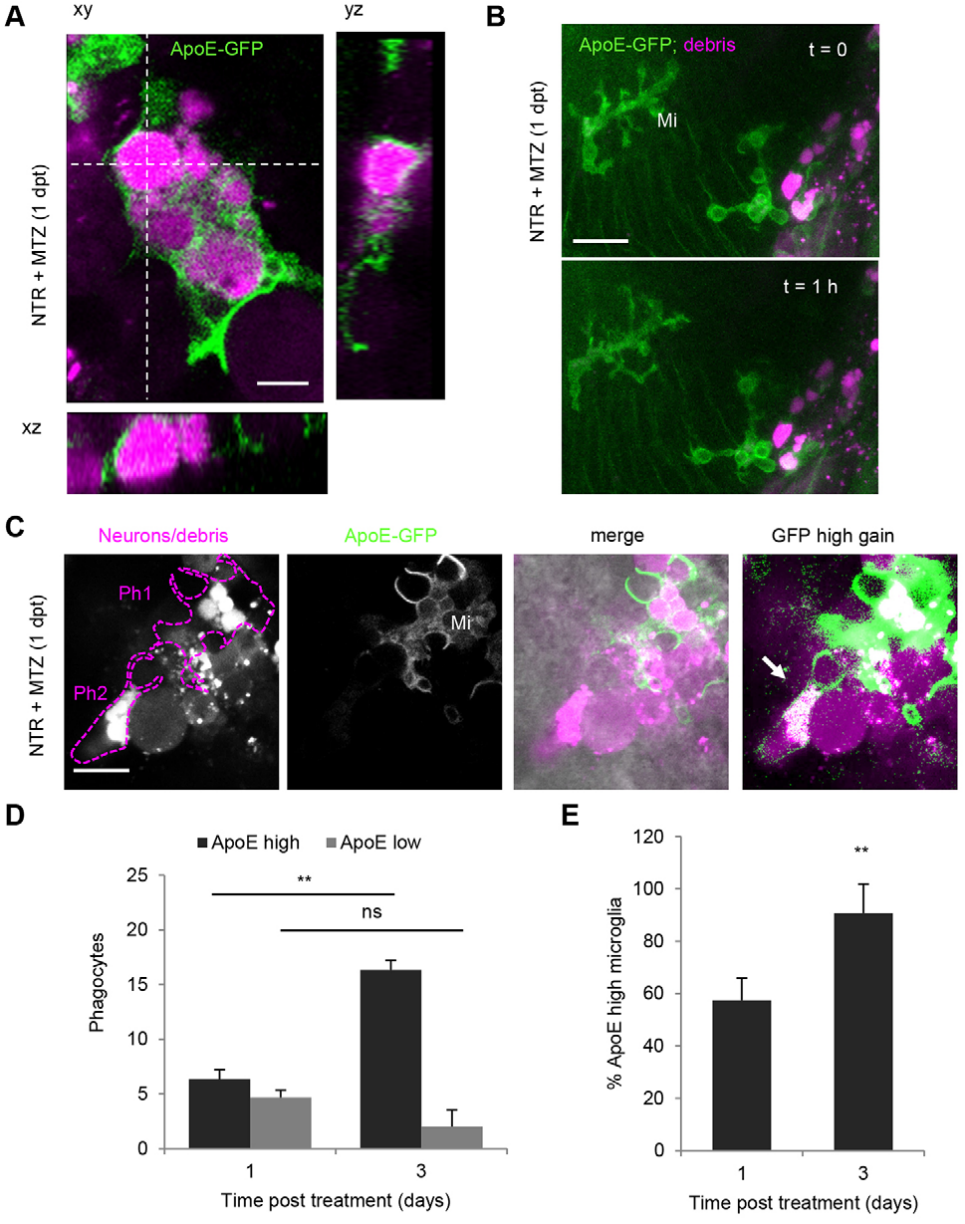
**Mononuclear phagocytes are ApoE-expressing microglia and macrophages.** (A) ApoE-driven membrane-targeted GFP expression in mCherry-positive phagocytes in NTR-ablated larval brain showing complete internalization of mCherry marked phagosomes.(B) Brain of NTR-ablated larva showing ramified ApoE-GFP microglia next to region of cell death and phagocytic macrophage and microglia. (C) Two mCherry-positive phagocytes in NTR-ablated larvae, one showing membranous ApoE-GFP expression and amoeboid morphology (Ph1), the other showing no membrane staining under normal imaging conditions (Ph2) (also see supplementary material Movies 3, 4). Upon high power excitation of GFP, a low level of ApoE-GFP-expressing population becomes apparent (arrow, right panel). (D) Number of phagocytes in the forebrain of NTR-ablated larvae and fraction coexpressing microglial ApoE-GFP at 1 and 3 days post-treatment. *Z*-stacks of 50–70 μm were used for quantification. (E) Fraction of ApoE-GFP-expressing microglia from total number of phagocytes 1 and 3 days post-ablation. Error bars indicate standard deviation; *n*=3 animals for 1 and 3 days post-treatment, respectively; **P≤0.01; ns, not significant. Mi, microglia. Scale bars: 5 μm (A), 20 μm (B), 10 μm (C). See also supplementary material Movies 3–6.

Apolipoprotein E-driven membrane-tagged GFP (ApoE-GFP) clearly labels the microglia subset of mononuclear phagocytes, and ApoE-expressing microglia are the only known phagocytes in the brain after about 3 days of development (Peri and Nüsslein-Volhard, 2008; Svahn et al., 2013). To elucidate whether microglia are involved in phagocytosis of damaged or injured neurons, transgenic animals expressing GFP in microglia were used. Half of the phagocytic cells in the brain showed a strong expression of ApoE-GFP at 1 day after ablation, indicating that these were microglia (Fig. 5A,C–E; supplementary material Movies 3–5). Upon imaging ApoE-GFP with higher laser power, we could detect the remaining phagocytic cell population, expressing low levels of GFP, which went unnoticed using conventional acquisition settings (Fig. 5C). This ApoE-GFP low-expressing (ApoE-low) population also showed phagocyte morphology, phagocytic cups and several red-fluorescent phagosomal structures (Fig. 5C, right panel). The microglia observed in degenerative areas were almost exclusively amoeboid, in line with their phagocytic activity (Fig. 5A,C; supplementary material Movies 3, 4). Remarkably, some ramified microglia were present within 50 μm of regions with dying cells and phagocytic cells (Fig. 5B; supplementary material Movie 6).

Over time, these microglia remained at a distance from the neurodegenerative area (supplementary material Movie 6), suggesting that some microglia exhibit a lack of (or a delayed) response to cell death compared with ApoE-low phagocytes. To address this possibility, the dynamics of the ratio of phagocytic microglia to ApoE-low phagocytes in the forebrain was determined. The numbers of ApoE-high microglia increased more than twofold between 1 and 3 days post-ablation (*P*≤0.005), whereas the numbers of ApoE-low phagocytes remained equal. The ratio of ApoE-high microglia to ApoE-low phagocytes increased from 57% at 1 day after ablation to 90% at 3 days after ablation (Fig. 5D,E; supplementary material Movie 5, *P*≤0.005). The increased proportion of microglia over time coincided with a relative decrease in ApoE-low phagocyte numbers, as numbers did not increase (Fig. 5E). Thus, microglia and ApoE-low phagocytes in forebrain increase in the first day after ablation. ApoE-low phagocytes peak early (~1 day) after ablation, after which their numbers neither increase nor decrease during the first 3 days after ablation. Microglial numbers continue to increase and numbers remain elevated for over a week. Therefore, ApoE-high microglia and ApoE-low phagocyte recruitment as well as decline show different temporal kinetics.

### Phagocytic cells are mpeg-expressing mononuclear phagocytes

**Fig. 6.**
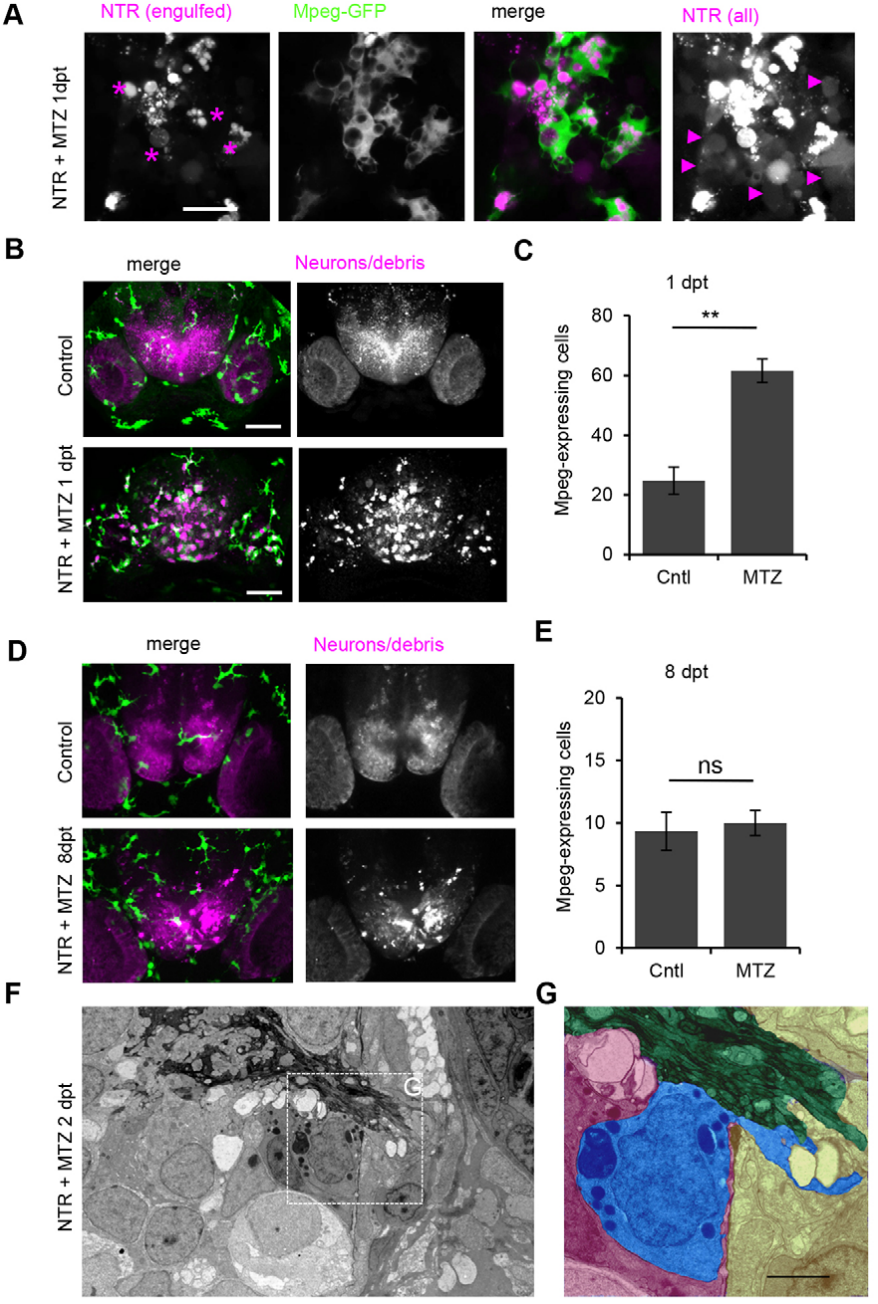
**Phagocytic responders are mononuclear phagocytes.** (A) Macrophage-expressed gene 1 (mpeg1)-driven GFP expression-positive phagocytes with amoeboid morphology in NTR-ablated larva 1 day post-treatment. (B) mpeg1-GFP expression in forebrain of control and treated larvae showing that mpeg1-positive (green) leukocytes co-localize with phagocytized debris in NTR animals treated with MTZ (also see supplementary material Movie 7). (C) Number of mpeg-expressing cells in control forebrain (*n*=4) and increased numbers in NTR MTZ-treated larvae (*n*=7) (*P*≤0.01). (D) mpeg-GFP expression in NTR control and treated larvae 8 days post-ablation. (E) Number of mpeg-expressing cells in control forebrain (*n*=3) and similar numbers in NTR larvae treated with MTZ (*n*=3) 8 days post-ablation, quantified from 80 μm *z*-stacks. (F) Electron micrograph of forebrain 2 days post-treatment showing olfactory nerve entering the brain. (G) False color high magnification view of phagocyte (blue) in F next to olfactory nerve (green) within meninges, with extended cytoplasmic process protruding outside of brain (blue). Error bars indicate standard deviation. **P≤0.01; ns, not significant. Scale bars: 10 μm (A), 50 μm (B,D), 2 μm (G). See also supplementary material Movies 7, 8.

Two types of mononuclear phagocytes have been characterized that express macrophage-expressed gene 1 (mpeg1)-GFP: peripheral macrophages and microglia (Ellett et al., 2011; Svahn et al., 2013). To address the dynamic response of mononuclear phagocytes upon neuronal cell death, mpeg1-driven GFP was introduced (supplementary material Table S1) (Ellett et al., 2011; Svahn et al., 2013). In control larvae, only a few ramified mpeg1-expressing cells were present in the forebrain, in line with previous studies (Svahn et al., 2013) and the L-plastin-positive cells (Fig. 6B, upper panels). One day after ablation, mpeg1-GFP-expressing cells were enriched in the forebrain (Fig. 6B, lower panels). In contrast to control larvae, these cells were spherical in shape (Fig. 6A). Furthermore, all fluorescent phagocytized material colocalized with mpeg1-GFP-expressing cells (Fig. 6A,B; supplementary material Movie 7). Additionally, phagocytic cell numbers in forebrains of NTR-ablated animals were increased more than twofold compared with controls at 1 day after ablation (Fig. 6B), in line with our findings using L-plastin (Fig. 2). Thus, all phagocytic cells present at the degenerative area of the CNS were mononuclear phagocytes.

The numbers of these phagocytes decline to basal levels 1 week after ablation (Fig. 1F). To examine whether this could be due to loss of mCherry fluorescent properties, ablated animals expressing mpeg1-GFP were imaged 8 days after ablation. Numbers of mpeg1-expressing cells in the rostral forebrain of NTR animals treated with MTZ were similar to those in wild-type animals (Fig. 6D,E). Thus, loss of mCherry fluorescence does not account for the decline in marked phagocytes, and 8 days post-treatment the numbers of mononuclear phagocytes in NTR-ablated brains had declined to control levels. Some of the mCherry-positive phagocytes were ApoE-high microglia (Fig. 5), which suggests that ApoE-low phagocytes in the brain could be peripherally recruited macrophages.

One mode of entry to the brain parenchyma for peripheral immune cells is by migration along the olfactory nerve fibers, through a porous bony plate called the cribriform plate, which separates the nasal cavity from the brain (Kaminski et al., 2012; Smithson and Kawaja, 2010). To address whether infiltration of peripheral macrophages along the olfactory nerve occurs in NTR zebrafish larvae treated with MTZ, large scale EM data were analyzed (Fig. 4). The data show that the meningeal layer enclosing the brain opens where the olfactory nerve bundles enter the brain. We found a phagocytic cell present within the brain that extended a long process outside of the brain through this gap (Fig. 6F,G). To further address whether this route is used for infiltrating macrophages *in vivo*, NTR larvae treated with MTZ expressing mpeg1-GFP were imaged at this particular area, early after onset of neurodegeneration. The data suggest that migration along the olfactory nerve allows mpeg1-GFP macrophages to directly enter the brain (Fig. 6F,G; supplementary material Movie 8). We conclude that two types of mpeg1-expressing mononuclear phagocytes are involved in clearance: microglial phagocytes showing high levels of ApoE-GFP and cells showing a very low level of ApoE-GFP expression. The latter might represent macrophages, which normally are not present in the brain and that could have entered the brain by migrating along the olfactory nerve (supplementary material Table S1).

### Apoptotic phagocytes are engulfed by microglia

**Fig. 7.**
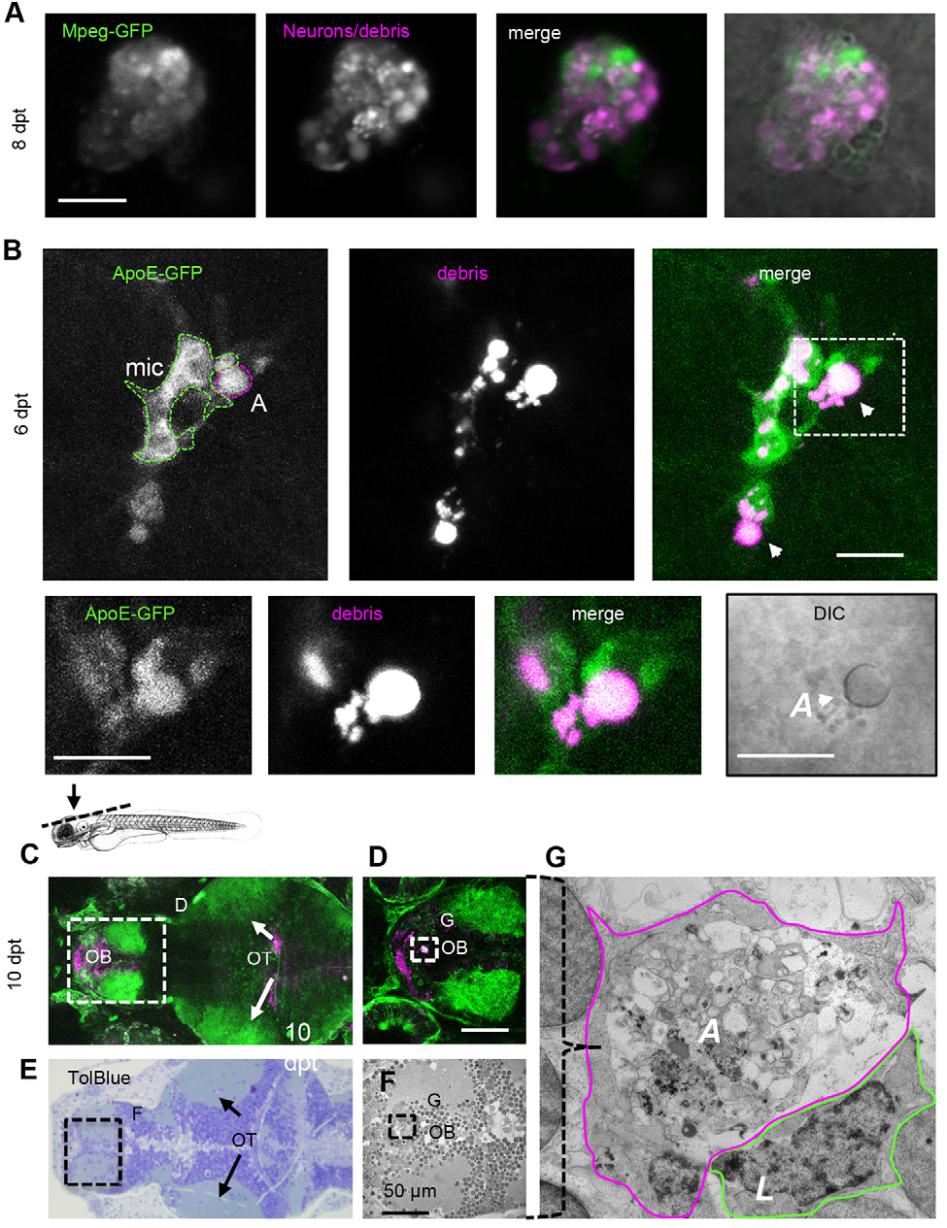
**Apoptotic phagocytes are engulfed by microglia.** (A) Large mpeg-GFP-expressing phagocyte 8 days post-treatment (dpt) showing cytoplasmic mCherry-positive vacuoles (magenta) and mpeg-GFP expression (green), suggestive of an apoptotic phagocyte. (B) ApoE-expressing microglia (mic; green) phagocytizing non-motile, apoptotic phagocytea (‘A’; magenta, arrowheads) (supplementary material Movie 9). The boxed area is shown below at higher magnification. The DIC image at the bottom right shows that the phagocyte being engulfed by microglia is apoptotic (reflective button-like rounded cell body). Data were recorded using intravital imaging of three distinct channels to visualize NTR, ApoE-GFP and apoptotic cells. The schematic above C indicates the region shown in C and E. (C) Optical section of live forebrain and optic tectum region of NTR animals treated with MTZ 10 days after treatment; green indicates GFP expression (supplementary material Movie 10). (D) Higher magnification of forebrain as shown in C. (E) Toluidine Blue stained section of region of brain of same animal as shown in C. (F) Electron micrograph of region indicated in E. (G) Electron micrograph of phagocytic profile observed in live imaging, showing large apoptotic cytoplasm including vacuoles and lysosomal debris demarcated with solid purple line, partly surrounded by a leukocytic cell (‘L’). ‘A’, apoptotic phagocyte; mic, microglia; OB, olfactory bulb; OT, optic tecti. Scale bars: 10 μm (A,B), 50 μm (D). See also supplementary material Movies 9–11.

Recovery of larvae a week after ablation suggests resolution of the immune response, which is often characterized by exit of leukocytes or programmed cell death of leukocytes followed by engulfment. As early as 1 day post-ablation many phagocytes were observed outside the CNS, suggesting their exit from the brain after engorging on dying cells (supplementary material Movie 1). Analysis of a few remaining phagocytes with ingested debris 8 days after ablation revealed that some cells were no longer migrating and were immobilized, sometimes for several days (Fig. 7A; supplementary material Movie 8, data not shown). When we analyzed these cells in mpeg1-GFP expressing animals, we found that they showed apoptotic morphology and cytoplasmic GFP expression (Fig. 7A). These phagocytes were rounded, lacking any dynamic appendages as seen in earlier phases (Fig. 7A). These cells in ApoE-GFP animals showed highly reflective, button-like objects, as identified with differential interference contrast microscopy (Fig. 7B, arrowheads), which is commonly used in *C. elegans* to reveal apoptosis (Sulston and Horvitz, 1977).

Intravital microscopy revealed that microglia often appeared immediately next to these large phagocytic cell corpses, attempting to engulf the phagocyte (Fig. 7A,B; supplementary material Movie 9) and occasionally succeeding (supplementary material Movie 10). Correlated microscopy (Giepmans, 2008) (EM analysis of the same cells previously tracked by fluorescent intravital imaging) further revealed that these were not neurons but probably phagocytes showing the typical morphology of the late stages of apoptosis (Fig. 7C–G; supplementary material Movie 11). These features included vacuoles, amorphous membranous structures and lack of dense cytoplasm found in phagocytic cells; the latter being characterized by the presence of many organelles (Fig. 7G; Fig. 4). Furthermore, we found a leukocyte next to the large structure, seemingly trying to phagocytose it, which was in line with our intravital microscopy data (Fig. 7G, indicated by ‘L’; supplementary material Movie 11). Therefore, a decline in leukocyte numbers is achieved through phagocytes exiting the central nervous system and by programmed cell death of the phagocytes and their engulfment by microglia.

## DISCUSSION

**Fig. 8.**
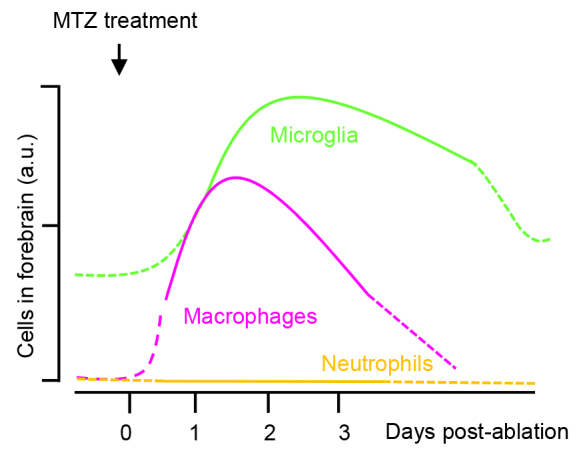
**Relative cell counts of dynamic differential leukocyte responses to cell death in the brain.** Schematic representation showing leukocyte recruitment dynamics based on data presented in the Results section.

Neurodegenerative diseases are generally chronic, and the age of onset is often not precise, which obscures identification of the dynamics of the cell types involved. Here, we identified the nature and timing of the phagocytic immune response to controlled cell death in the larval zebrafish brain, employing the benefits of advanced microscopy approaches. In our working model (Fig. 8), the only two types of immune cells involved are microglia and recruited peripheral macrophage-like cells. Interestingly, ApoE-high and ApoE-low phagocytes show different temporal kinetics. The response can be divided into three phases: (i) There is a recruitment phase during the first day following neuronal damage, when phagocyte numbers increase; (ii) In the second phase, microglial numbers continue to go up, whereas the ApoE-low-expressing phagocytes largely disappear from the forebrain within 3 days of ablation; (iii) In the final phase, microglial numbers decline to baseline about 8 days after ablation.

### Immune cells control tissue repair

Remarkably, most NTR-ablated animals survived to adulthood, and adults that received neuronal ablation at larval stages show normal vital functions such as feeding and mating behavior. Additionally, our histological analysis of brains a month after ablation showed no evidence of pathological features, loss of cells or other lesions. Ablation mainly affects the nose and olfactory bulb, which are required for behaviors ranging from feeding to courtship (Kermen et al., 2013). We did not observe alterations in any of these behaviors, which implies functional recovery of the affected brain tissue. Therefore, the immune responses we observe probably do not impair nervous tissue repair, but rather contribute to the repair process. A role for mononuclear phagocytes in tissue repair is not unlikely because, after neural injury and in several brain disease models, specific infiltrating macrophages have anti-inflammatory and reparative properties, and immune infiltrate is required for regeneration in zebrafish brain following forebrain stab wounds (Kyritsis et al., 2012; London et al., 2013). Furthermore, limb regrowth in salamanders is specifically dependent on the presence of macrophages (Godwin et al., 2013). Thus, cellular immune responses can be a prerequisite for tissue regeneration. At the same time, infiltrating monocyte-derived macrophages correlate well with tissue damage in the multiple sclerosis EAE model and meningitis mouse models. Therefore, whether these cells have a beneficial or detrimental effect is probably disease-dependent (Ajami et al., 2011; Kim et al., 2009). Of note, zebrafish show high regenerative capacity of tissues that show notoriously little regenerative capacity in mammals, such as cardiac and brain tissue (Kroehne et al., 2011; Poss et al., 2002). Therefore, identifying the conditions that promote regeneration of zebrafish neuronal tissues might help understand the low regenerative potential in mammals (Powell et al., 2013).

### Clearance is performed by two types of cells

We only find L-plastin-positive cells (leukocytes) involved in clearance, suggesting that potentially phagocytic glial cells such as radial glia (the zebrafish counterparts of astrocytes) are not involved in engulfment and phagocytosis of cell debris in this model (Cahoy et al., 2008). We also show that neutrophil granulocytes are not involved because they remain absent from the brain following ablation, suggesting that programmed cell death in the brain fails to attract granulocytes. Although granulocytes are absent in the CNS under physiological conditions, in necrotic lesions like a damaged blood brain barrier or cerebral infection these cells can infiltrate neural tissue (Dirnagl et al., 1999). In zebrafish, granulocytes also infiltrate neural tissue upon ventricular bacterial injection, spinal cord lesions or systemic inflammation caused by a recently discovered mutant showing defective microglial deployment (Goldshmit et al., 2012; Hall et al., 2012; Renshaw et al., 2006; Shiau et al., 2013). Therefore, we argue that chemotaxis of granulocytes or entry of granulocytes to the brain is not induced upon programmed cell death in the brain. Engulfment following neuronal cell ablation is therefore restricted to microglia and infiltrating macrophages and thus does not involve astrocytes or granulocytes.

A previous study on zebrafish microglia showed direct chemotaxis of microglia in response to localized acute laser-mediated cell death, and did not show involvement of other leukocyte classes (Sieger et al., 2012). However, as this was not the focus of the study it is unclear whether other cell types were involved. Different types of neuronal damage probably elicit different types of cues that attract immune cells, and it will be interesting to compare immune cell behavior after different types of neuronal damage to better understand these cues.

No phagocytes except for ApoE-expressing microglia have been found in zebrafish brain at the stages we investigated under physiological circumstances (Herbomel et al., 2001; Peri and Nüsslein-Volhard, 2008). However, we found, in addition to activated resident ApoE-high microglia, a second mononuclear phagocyte cell type that expressed hard-to-detect levels of ApoE. At least some of these ApoE-low cells are peripherally recruited macrophages, as indicated by our data showing that macrophages can infiltrate the brain under these conditions. This suggests that programmed cell death in the brain attracts peripheral mononuclear phagocytes. Based on morphology, behavior and marker analysis, these macrophage-like cells could represent monocyte-derived macrophages, although at this stage we cannot exclude the possibility that dendritic cells are also attracted or the presence of a previously undefined subset of microglia. In fact, it is possible that some of the ApoE-low phagocytes might represent a microglial-subtype not marked by the ApoE-transgenic line.

Despite a lack of obvious functional differences between the two cell types, they showed different temporal kinetics, with the macrophage-like cells mostly present in early stages, followed by microglia. Because some microglia are ramified and non-activated at those stages, macrophage-like cells might be more prone to respond in early stages, for example through a higher expression of receptors causing their recruitment. Simultaneously, there might be regional diversity of microglial phenotypes, and some brain areas might be more accessible to peripheral leukocytes (de Haas et al., 2008; Scheffel et al., 2012). Interestingly, in the mouse EAE multiple sclerosis model, microglial activation occurs prior to monocyte-derived macrophage recruitment, suggesting that the kinetics are disease-cue dependent (Ajami et al., 2011). An alternative explanation for our observation of an increasing fraction of ApoE-high expressing phagocytes is that ApoE expression in low-expressing cells increases over time. We did not observe such an increase in ApoE expression in phagocytes in our long-term live imaging experiments. It is unclear at this stage why under these conditions peripheral macrophage-like cells invade the brain upon neuronal ablation, and what causes their differential activation. A partial explanation is that numbers of microglia increase strongly after 9 dpf in zebrafish (Svahn et al., 2013), suggesting that the phagocytic capacity at stages examined in this study might be quite low. Because macrophage infiltration and microglial activity show differential disease-dependent patterns of involvement, we argue that these studies in zebrafish could open up avenues for dissecting the mechanisms responsible for differential involvement of immune cells in brain disease.

### Restricted access of mononuclear phagocytes into the zebrafish brain

Only specific subclasses of leukocytes infiltrate the CNS under ablation conditions, suggesting that accessibility or recruitment of these cells in larval zebrafish brain to infiltration by immune cells is selective and regulated. Entry routes from the blood circulation in mammals could include migration across the wall of blood vessels in post-capillary venules or across the blood-cerebrospinal fluid barrier (Ransohoff et al., 2003; Shechter et al., 2013). Although our results show that macrophage infiltration of the brain parenchyma can occur by migrating of macrophages along the olfactory nerve, as previously shown in mouse models, at this stage it is not clear whether there is a preferred pathway (Kaminski et al., 2012; Smithson and Kawaja, 2010).

### Apoptosis and engulfment of phagocytes during the resolution phase

The phagocytic cells that remain in the CNS undergo programmed cell death and are sometimes engulfed by microglia. Although apoptotic microglia have been described in animal models as well as in human brain tissue, the fate of such cells is unclear. In peripheral wounding responses in vertebrates, recruited immune cells are generally short-lived and succumb to programmed cell death, a phenomenon known as resolution of inflammation (Martin and Leibovich, 2005; Serhan and Savill, 2005). Infiltrating monocyte-derived macrophages are also short-lived (Jeong et al., 2013; Shechter and Schwartz, 2013a). Our data in zebrafish suggest that recruited mononuclear phagocytes as well as microglia undergo apoptosis and are engulfed by microglia. If this process is conserved in mammals, this would suggest that programmed cell death of phagocytes and subsequent engulfment might serve to resolve neuroinflammation in the brain. Therefore, similarly to wound healing, resolution of inflammation in larval zebrafish brain is achieved by migration of immune cells away from the injured site and engulfment of macrophages by microglia. The latter finding provides a novel cellular mechanism whereby inflammation can be resolved, which might be relevant to the understanding of brain diseases involving chronic inflammation.

## MATERIALS AND METHODS

### Zebrafish culture

Zebrafish (mix of AB and longfins) were reared according to standard conditions (Kimmel et al., 1995). Embryos were initially raised at 28°C on a 14–10 hour light-dark cycle in 50% system water containing Methylene Blue. During experiments, larvae were kept in HEPES buffered (pH 7.2) E3 media as described (Kimmel et al., 1995). Larvae used for experiments were grown in medium containing 1-phenyl 2-thiourea (0.003%) to prevent pigment formation. For survival analysis, larvae were fed paramecia starting at 7–8 dpf. Animal experiments were approved by the Animal Experimentation Committee of the University of Groningen.

### Whole mount immunofluorescence histochemistry and TUNEL labeling

L-plastin immunohistochemistry was performed basically as described (Redd et al., 2006). Briefly, larvae were anesthetized and fixed in fresh 4% paraformaldehyde (PFA) in PBS containing 0.4% Triton X-100 (PBSTx) for 1–2 days at 4°C. Samples were dehydrated in 70% ethanol overnight, blocked for 2 h at room temperature in PBSTx containing 1% BSA and 0.1% DMSO. Larvae were incubated with PBSTx containing 5% BSA and the primary antibody (antibodies) with gentle shaking at 4°C overnight, after which they were washed extensively. Rabbit L-plastin anibody was used to label all leukocytes (a gift from Yi Feng and Paul Martin, Bristol). Larvae were incubated with secondary antibodies in PBST containing 2% BSA (Jackson, DyLight, 488 donkey anti-rabbit) at 4°C overnight. Samples were rinsed and then washed extensively. Larvae were imaged whole mount in 1.8% LMP agarose in 6-cm dishes for upright and in 2.5-cm MatTek dishes for inverted microscopy, as described for *in vivo* imaging (van Ham et al., 2012).

TUNEL staining was performed basically as described (van Ham et al., 2010). Animals were fixed as described above, treated with proteinase K (10 μg/ml) in PBST for 40 minutes and re-fixed in PFA. The TUNEL assay was visualized using the Click-iT TUNEL Alexa Fluor 647 imaging assay kit (Life Technologies). Animals were rinsed in PBST containing 3% BSA after TdT and Click-iT incubations. Imaging of mCherry and Alexa Fluor 647 fluorescence was performed by excitation using 561 and 633 nm laser lines, correspondingly.

### Correlated and large scale scanning transmission electron microscopy

Sample fixation and processing procedures were performed basically as described (Schnell et al., 2012). Briefly, larvae were fixed for 2 hours in PBS containing 4% PFA and 0.05% Triton X-100 (and subsequently in fixative containing 0.5% PFA, 2% glutaraldehyde and 0.1 M cacodylate, pH 7.4) overnight at 4°C. Samples were rinsed twice in PBS, and heads were sectioned rostrally to the hindbrain to facilitate penetration of osmium. Fixed larvae were postfixed in 1% osmium tetroxide (OsO_4_), 1.5% potassium ferrocyanide (K_4_[Fe(CN)]_6_) on ice for 2 hours, and dehydrated in ethanol. Subsequently, larvae were incubated overnight in EPON 1:1 mixed with 100% ethanol, before embedding in EPON. EPON blocks were sectioned using an ultramicrotome (Ultracut E, Reichert-Jung and Leica). Semithin sections were stained using Toluidine Blue or Fuchsin as described. Ultrathin sections were mounted on Formvar-coated nickel grids and stained with lead citrate and uranyl acetate. Transmission electron microscopy (TEM) images were recorded using a FEI CM100 operating at 80 kV equipped with a Morada digital camera (Olympus SIS, Germany). For large-scale EM, acquisition was different to that previously described when we used camera-based recording using TEM (Ravelli et al., 2013). Here, we used scanning transmission electron microscopy (STEM), which allows generation of a large field of view at high resolution. Typically, one STEM image is equivalent to the fields of view of 50–100 TEM images, significantly reducing the amount of stitching when imaging large fields of view at high resolution. Ultrathin sections were mounted on one-hole grids and scanned using a Zeiss Supra 55 scanning electron microscope in STEM mode with an ATLAS external scan generator and software (Fibics, Canada). Images were recorded with 2 nm pixel size at 29 kV. Stitching was performed using VE-viewer (Fibics). Analysis was performed using ATLAS browser-based viewer Zeiss (Fibics, Canada), Photoshop CS5.1 (Adobe, USA) and Fiji (ImageJ, USA). For correlative EM, zebrafish were imaged using confocal or two-photon imaging in agarose as described. After acquiring imaging data, larvae were fixed in agarose using 4% PFA in PBSTx, and imaged again. Subsequently, larvae were cut from agarose and processed as described for large-scale EM. Known anatomic nervous structures in the brain, including gray-white matter boundaries, were used to identify the region of interest during ultrathin sectioning.

### Intravital imaging

Zebrafish were mounted in 1.8% low melting point agarose containing tricaine in HEPES-buffered E3 as described. Imaging was performed in a heated chamber at ~28°C using Zeiss LSM780 and 7 MP confocal and multiphoton systems (Zeiss). For multicolor two-photon microscopy of red (mCherry) and green (GFP) fluorophores the laser (Coherent, Santa Clara, CA) was tuned to ~970 nm (940–970 nm) and 500–550 and 575–610 filter sets were used. A 20× dipping objective (Zeiss W Plan A 20×, 1.0 NA) was used for these experiments. Multicolor confocal microscopy was carried out on a LSM780 system equipped with a transmission detector for DIC using Zen software. PlnApo 20×, 0.8 NA DICII and Lcl Plan Neofluar 63×, 1.3 NA lenses were used and 405, 488, 514, 561, 594 and 633 laser lines. An automated stage was used on both 7 MP and LSM780 systems to acquire multiple time-lapse recordings simultaneously, and acquire *x*-*y* tile scans and *z*-stacks of each of these animals. For imaging of mpeg-GFP transgenic animals, an LSM700 system (Zeiss) was used as described (van Ham et al., 2012). For 3D analysis of phagocytes, a 63× high numerical aperture lens was used and Zen optimized *z*-stack intervals to acquire optimal resolution in the *z*-dimension.

### Adult brain histology

NTR zebrafish treated with MTZ and control zebrafish were allowed to recover up for several weeks. To dissect their brains, animals were first anesthetized on ice, and heads were severed behind the gills. The lower jaw, gills and eyes were removed using watchmaker’s forceps and brains were fixed inside the skull in Zamboni fixative (4% PFA, 2% glutaraldehyde, 0.2% picric acid in 0.1 M sodium cacodylate, pH 7.4) overnight at 4°C. Skulls containing whole brains were rinsed twice in 0.1 M sodium cacodylate and dissected from the skull using forceps (#5, Dumont) and a 20-gauge hypodermic needle. Images of fixed zebrafish brains were acquired using a Leica fluorescence dissection microscope. Subsequently, osmium fixation and embedding in EPON of whole brains was performed as described for electron microscopy. Sections were stained using Toluidine Blue and basic Fuchsine and visualized using an Olympus microscope.

### NTR experiments

Zebrafish were treated with MTZ as described (van Ham et al., 2012). Briefly, for ablation experiments, animals at specified days (5 dpf, except for Fig. 1B,C where treatment was performed at 2 dpf) were treated overnight (16 hours) with MTZ dissolved in DMSO after which they were rinsed extensively in E3 buffer to dilute MTZ over 1000-fold compared with the starting concentration. The amount of cell death caused by 2 mM MTZ was sufficient to elicit a strong immune response but low enough to ensure reversibility of the injury and levels of survival to adulthood similar to those of wild-type animals. For recovery assays (lifespans) animals were kept at 28°C on a 14–10 hour light-dark cycle in 50% system water. After ~8 dpf, larvae were fed paramecia daily. Numbers of animals alive were counted every day after induction of cell ablation.

### Transgenic lines

Transgenic lines used in this study were combinations between MPX-GFP (Renshaw et al., 2006) and MPEG-GFP (Ellett et al., 2011). For NTR experiments, UAS-NTR/mCherry; NeuroG4-mCherry double transgenic animals, ApoE-GFP and PU.1-GFP transgenic animals were used as described previously (van Ham et al., 2012).

### Chemicals

Metronidazole (Sigma), tricaine methanesulfonate (MS222) (Sigma), *N*-phenylthiourea (PTU, Sigma), low melting point (LMP) agarose (Sigma).

### Statistics analysis

Statistical significance was calculated using the Student’s *t*-test. For image processing and quantitative analysis, Excel (Microsoft), Photoshop (Adobe), Prism (Graphpad), Fiji ImageJ, Zen (Zeiss) and Imaris (Bitplane) were used.

## Supplementary Material

Supplementary Material
