# Supplementary material for: Intravital correlated microscopy reveals differential macrophage and microglial dynamics during resolution of neuroinflammation
Source: Dis Model Mech. 2014 Jul;7(7):857–69. doi: 10.1242/dmm.014886 (PMC4073275; doi:10.1242/dmm.014886)
Supplement: Supplementary Material [file supp_7_7_857__index.html]

Intravital correlated microscopy reveals differential macrophage and microglial dynamics during resolution of neuroinflammation — Supplementary Material 

# Intravital correlated microscopy reveals differential macrophage and microglial dynamics during resolution of neuroinflammation

## DMM014886 Supplementary Material

**Files in this Data Supplement:**

- **Supplementary Material**
