# Supplementary material for: Intravital correlated microscopy reveals differential macrophage and microglial dynamics during resolution of neuroinflammation
Source: Dis Model Mech. 2014 Jul;7(7):857–69. doi: 10.1242/dmm.014886 (PMC4073275; doi:10.1242/dmm.014886)
Supplement: Supplementary Material [file supp_7.7.857_DMM014886.pdf]

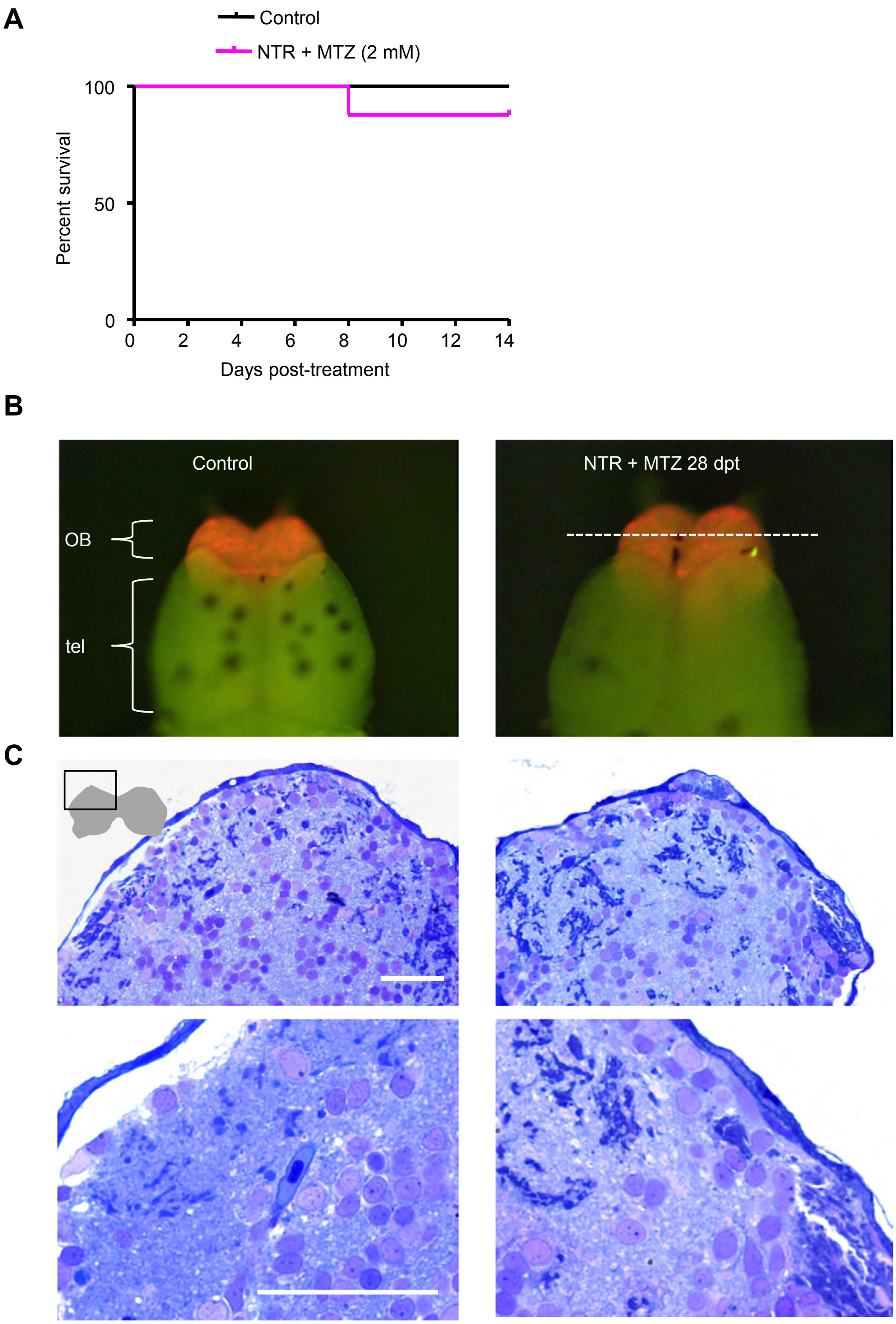

Figure S1. Related to Fig. 1. (A) Survival over 2 weeks of control and neuro-NTR larvae after metronidazole treatment. Larvae are treated with 2 mM metronidazole (MTZ) at 5 days post fertilization. MTZ is washed out after overnight (16 h) incubation. These larvae are allowed to grow up initially in petri dishes at 28ºC for 2 days post treatment, and in small tanks afterwards during which they are fed paramecia daily. Control animals (n=44), NTR larvae treated with MTZ (n=33). (B) Dissected control and NTR treated brain 4 weeks post-treatment showing mCherry fluorescence in the olfactory bulb. (C) Toluidene blue stained sections of control and NTR treated brains shown in (B). Region shown is indicated by schematic representation of the two lobes of the olfactory bulb. OB indicates olfactory bulb, Tel indicates telencephalon.

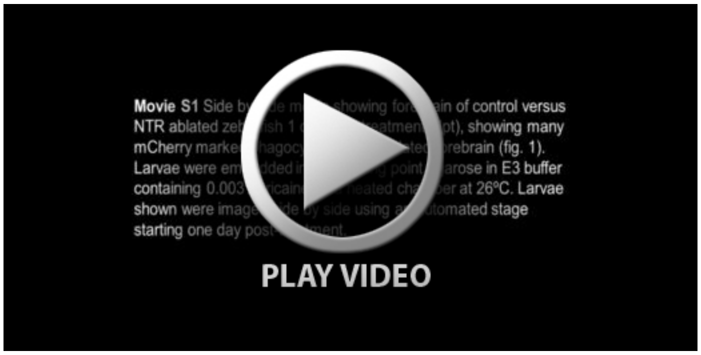

Movie 1. Related to Fig. 1. Controlled ablation of brain cells is followed by phagocytosis. 2-photon imaging of ablation 1 dpt. Side by side movie showing forebrain of control versus NTR-ablated zebrafish 1 day post treatment. NTR expressing neurons (white) are shown mostly in control, mCherry marked phagocytes (white) in NTR ablated forebrain (Fig. 1). Larvae shown were imaged side by side using an automated stage starting one day post-treatment. Data was acquired using a 2 photon laser scanning microscope (7MP; Carl Zeiss, Inc.). Z-stacks were acquired every 10 minutes.

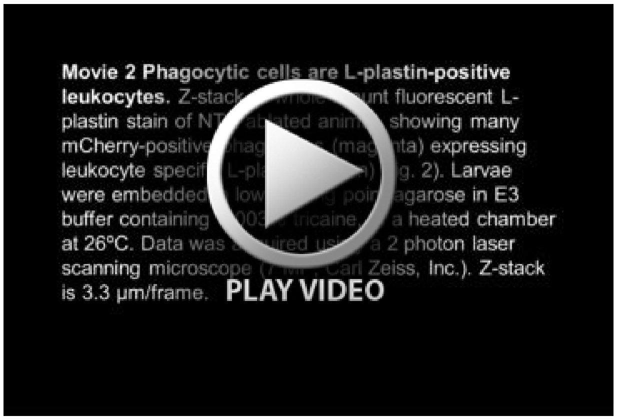

Movie 2. Related to Fig. 2. Phagocytic cells are L-plastin-positive leukocytes. Z-stack of whole mount fluorescent L-plastin stain of NTR ablated animal, showing many mCherry-positive phagocytes (magenta) expressing leukocyte specific L-plastin (green) (Fig. 2). Z-stack is 3.3 µm/frame.

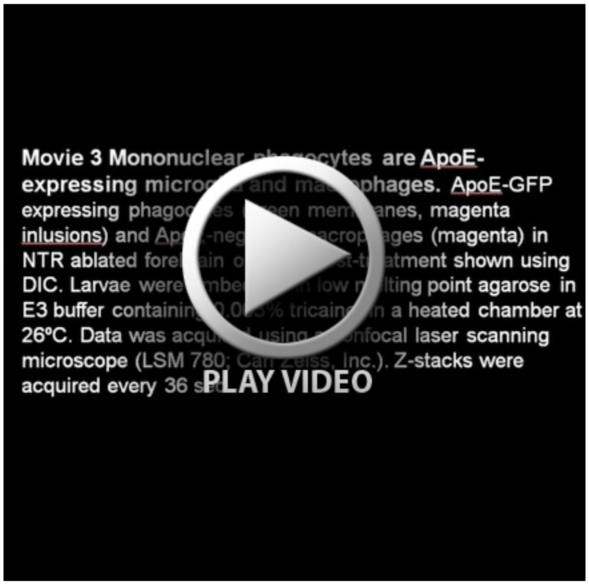

Movie S3. R elated to Fig. 5. Phagocytes are ApoE-expressing microglia and macrophages. ApoE-GFP expressing phagocytes (green membranes, magenta inclusions) and ApoE-negative macrophages (magenta) in NTR ablated forebrain one day post-treatment. Z-stacks were acquired every 36 seconds.

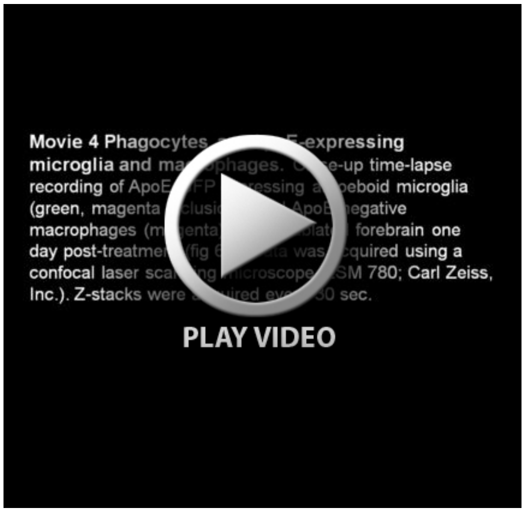

Movie S4. Related to Fig. 5. Phagocytes are ApoE-high microglia and ApoE-low phagocytes. Close-up time-lapse recording of ApoE-high amoeboid microglia (green, magenta inclusions) and ApoE-low phagocytes (magenta) in NTR ablated forebrain one day post-treatment (Fig. 5B). Z-stacks were acquired every 30 seconds.

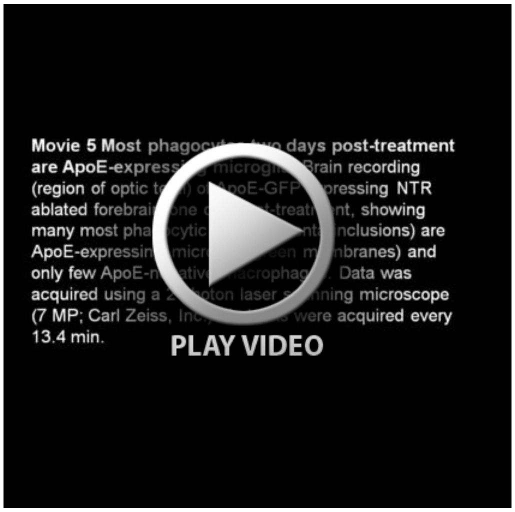

Movie 5. Related to Fig. 5. Most phagocytes two days post-treatment are ApoEexpressing microglia. Brain recording (region of optic tecti) of ApoE-GFP expressing NTR ablated forebrain one day post-treatment, showing many most phagocytic cells (magenta inclusions) are ApoE-expressing microglia (green membranes) and only few ApoE-low phagocytes. Z-stacks were acquired every 13.4 minutes.

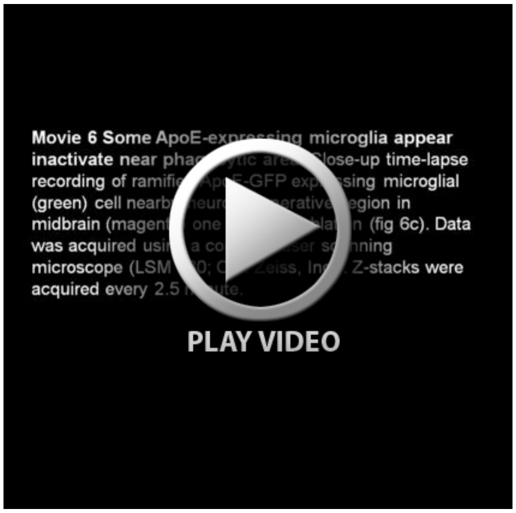

Movie 6. Related to Fig. 5, Some ApoE-expressing microglia appear inactivate near phagocytic area. Close-up time-lapse recording of ramified ApoE-GFP expressing microglial (green) cell nearby neurodegenerative region in midbrain (magenta) one day post-ablation (Fig. 6C). Z-stacks were acquired every 2.5 minutes.

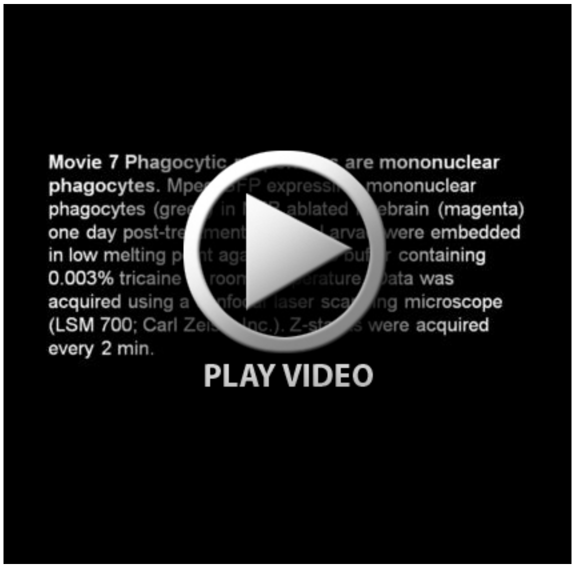

Movie 7. Related to Fig. 6. Phagocytic responders are mononuclear phagocytes. Mpeg1-GFP expressing mononuclear phagocytes (green) in NTR ablated forebrain (magenta) one day post-treatment (Fig. 6). Z-stacks were acquired every 2 minutes.

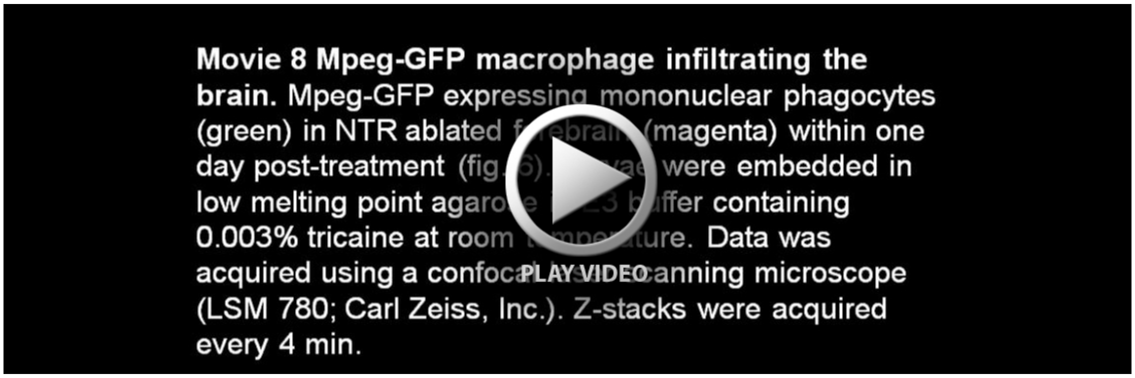

Movie 8. Related to Fig. 6, Mpeg-GFP expressing macrophage entering the forebrain through. Mpeg1-GFP expressing mononuclear phagocytes (green) in NTR ablated forebrain (magenta) within a day post-treatment (Fig. 6). Z-stacks were acquired every 4 minutes.

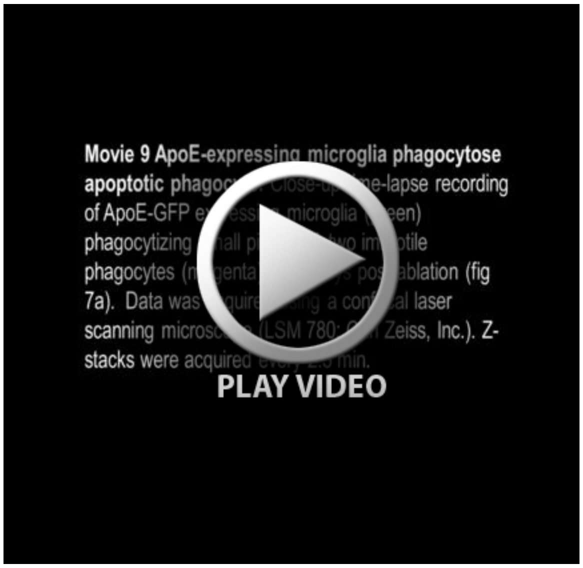

Movie 9. Related to Fig. 7. ApoE-expressing microglia phagocytose apoptotic phagocyte. Close-up time-lapse recording of ApoE-GFP expressing microglia (green) phagocytizing small pieces of two immotile phagocytes (magenta) six days postablation (Fig. 7B). Z-stacks were acquired every 2.5 minutes.

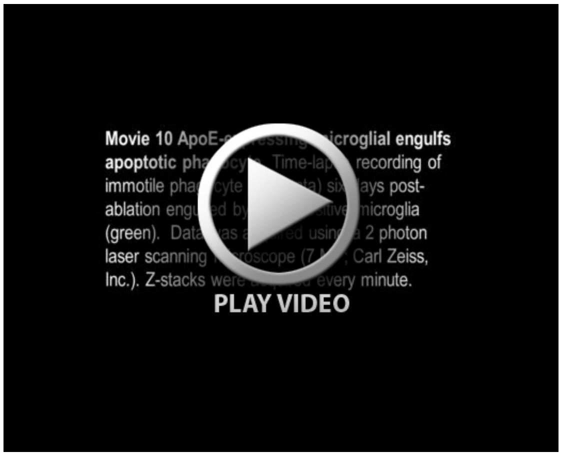

Movie 10. Related to Fig. 7. ApoE-expressing microglial engulfs apoptotic phagocyte. Time-lapse recording of immotile phagocyte (magenta) six days post-ablation engulfed by ApoE positive microglia (green). Z-stacks were acquired every minute.

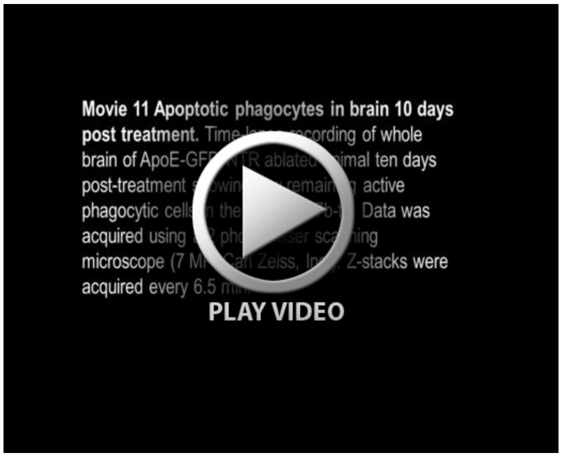

Movie 11. Related to Fig. 7. Apoptotic phagocytes in brain 10 days post treatment. Time-lapse recording of whole brain of ApoE-GFP, NTR ablated animal ten days posttreatment showing few remaining active phagocytic cells in the brain (Fig. 7C-G). Zstacks were acquired every 6.5 minutes.

**Expression**	**Immune cell type**		
	**Neutrophils**	**Mononuclear phagocytes**	
		**Microglia**	**Macrophages**
L-plastin	+	+	+
MPX	+	-	-
ApoE	-	+	-
Mpeg	-	+	+

Table S1. **Related to Fig. 1-6.** Fluorescent markers used for four different expressed genes to distinguish three types of leukocytes. These markers are complemented by morphological, behavioral and ultrastructural criteria.
